# AI image enhancement for failure analysis in 3D quantum information technology

**DOI:** 10.1038/s41598-025-08308-4

**Published:** 2025-07-05

**Authors:** Raphael Wilhelmer, Fabian Laurent, Tatjana Djuric-Rissner, Max Glantschnig, Johann Strasser, Stefan Weinberger, Tobias Herrmann, Clemens Rössler, Peter Czurratis, Roland Brunner

**Affiliations:** 1 Materials Center Leoben Forschung GmbH, Leoben, Austria; 2 Infineon Technologies AG, Villach, Austria; 3 PVA TePla Analytical Systems GmbH, Westhausen, Germany; 4 Infineon Technologies AG, Regensburg, Germany

**Keywords:** Electrical and electronic engineering, Imaging techniques

## Abstract

3D integration and miniaturization techniques get more widely used in conventional integrated circuits but also represent crucial ingredients for future quantum computing devices. This consolidates the need for efficiently detecting increasingly small defects on wafer size. Here we present a time-efficient and accurate way of measuring, localizing and statistically classifying defects down to the micrometer regime, utilizing a combination of scanning acoustic microscopy (SAM), You Only Look Once object detection, semantic segmentation and a machine learning super-resolution (ML-SR) approach. In particular, we test the capabilities of different ML-SR approaches to enable self-supervised quality enhancement of the measured image data. We reveal that the developed AI-powered workflow enhances time-efficiency by a factor of around 4x and 6x for the TSV and delamination analysis, respectively. Yet, the mentioned approach is not limited to SAM image data but presents a general way for speeding-up failure analysis in various fields.

**Supplementary Information:**

The online version contains supplementary material available at 10.1038/s41598-025-08308-4.

## Introduction

Parallel to the trend of miniaturization and high-density integrated circuit (IC) technologies in classical computation, quantum computing (QC) provides another emerging field harnessing quantum mechanical instead of classical binary algorithms^1^. Hence, QC offers the possibility of solving computational problems beyond the ability of the most powerful classical computers. Quantum computers are proposed to perform especially well in cases where optimization problems play a large role, like in modelling molecular energy levels^2–4^, machine learning^5–7^, etc. In order to achieve this quantum supremacy, several quantum bit (qubit) implementations are discussed in research but also in context to industrial applications. Approaches include the use of vibrating molecules^8^, resonating superconductors^6,9^, spin qubits^10–12^, photons^1,7^, atoms^13,14^ or ion traps^15,16^. However, ultimately for the establishment of QC into the society, qubit up-scaling displays an essential ingredient. At this point ion traps, in particular, offer great potential not only due to high confinement forces, as well as unparalleled control at the single-atom level, but also by allowing high scalability by being CMOS fabrication compatible^16,17^.

Even though several recent advancements in lab-based quantum technologies emerged, challenges remain towards industrialization in context to integration and miniaturization^15,18,19^. 3D integration technology and miniaturization techniques have evolved rapidly in recent years for classical microelectronic devices to retain Moore’s law^1,20^ and enable the development of higher-density circuits with improved performance and efficiency^21,22^. The application of 3D integration techniques not only brings benefits to classical hardware architectures but also to QC technologies utilizing silicon wafer platforms^18,23^. It offers denser integration of qubits, improved control for the electronics as well as exhibits reduced wiring^24^. In general, wafer-to-wafer bonding^25–27^ displays a 3D integration technology for stacking multiple heterogenous chips with high 3D interconnect densities. Of special importance in this context are vertical electrical connections passing completely through the silicon wafers or dies. Electrical interconnects, such as through-silicon vias (TSVs), enable the reduction of signal-delays and power consumption^28,29^.

Nevertheless, to meet the unique requirements of QC, wafer bonding- and TSV-technologies have to be adapted. This is, however, not without challenges, since bonding and TSV production processes are delicate and small imperfections during electroplating, etching or oxide-removal can lead to the failure of the whole device^30,31^. TSVs, for example, show various defect types including voids resulting from electroplating^31^, adhesion problems arising due to thermal expansion mismatch^28^ or cracks resulting from global stress in the die warpage^28,32^. Wafer bonding techniques, on the other hand, mainly suffer from failure generation due to insufficient adhesion properties between the two bonded wafers or void formation^26,27,33^. Hence, there is a need for fast, accurate and, above all, automated failure analysis workflows suitable to operate non-destructively on wafer-level with the ability to collect statistically relevant information, and ultimately to enhance production and device quality with reduced device failure.

Scanning Acoustic Microscopy (SAM) offers an efficient method to carry out non-destructive failure analysis in the field of microelectronics and power semiconductors^34–36^. The achievable resolution and contrast of SAM is restricted by the utilized frequency of the acoustic waves and the number of pixels to be resolved^37,38^. Increasing the number of scanned pixels enables the detection of smaller defects, however goes also with an increased scanning time. Therefore, the scanning of large areas incorporating the whole wafer area with high resolution and contrast is time-consuming and costly. Additionally, a lower practical limit is set to around several hundred nanometers by the physical properties of high frequency acoustic waves, which are absorbed by the coupling medium before penetrating into or reaching the sample^37,38^.

To solve the problem of long scanning times at high resolution, image enhancement approaches^39^ can be utilized. Whereas classical methods like bicubic- or spline-upscaling are a possibility for increasing resolution, they often perform mediocre in terms of restoring image quality^40^. A much more capable way of increasing image size is by the use of machine learning (ML)-based super-resolution algorithms. Super-resolution is a strongly discussed topic in literature dealing with imaging, and several different concepts have been proposed^40–43^. The discussed approaches reach from simple but capable convolutional neural networks (CNNs)^42^ and generative adversarial networks (GANs)^41^ to the impressive results of current generative diffusion^44^ or diffusion-like^43^ models. GANs work by combining two models, a generator and a discriminator. The generators job is to generate high-resolution images from low-resolution images, whereas the discriminator tries to detect these fake high-resolution images^40,41^. Instead of predicting the HR image in one go, like CNNs or GANs do, diffusion models use an iterative procedure to increase image fidelity step by step, also avoiding regression-to-the-mean^43,45–47^. However, most state-of-the-art super-resolution models need paired low- and high-resolution images for training and are not capable of being applied to out-of-domain real-world data^48^. Only very few super-resolution models, mainly based on self-supervised learning, tackle these problems^49,50^. Furthermore, the consideration of generative artificial intelligence hallucinations, creating a model output that is either nonsensical or false, is crucial for the selection of the model^51,52^. Onward, also energy consumption in combination with model performance are important selection criteria.

For the subsequent statistical defect analysis, object detection or segmentation algorithms can be employed^53^. Early ML-based methods utilize Random Forest and K-Nearest Neighbors algorithms for object detection^34^. In contrast to these methods, the recent sliding window technique, utilized within a convolutional neural network (CNN), shows great advantages for TSV localization in terms of speed and accuracy^34^. Even faster object detection can be performed by one-shot methods like You Only Look Once (YOLO)^54,55^ or transformer-based models^56^. For the segmentation of relevant features, various classical approaches like Canny Edge Detection^57^, Fringe Segmentation Techniques^34^ or thresholding as well as ML-based algorithm^58^ to process large amounts of image data, can be applied. However, all mentioned methods have problems in detecting objects close to the resolution limit, usually resulting in low localization, classification and segmentation accuracy^59^.

In this paper, we introduce a fully automated image analysis workflow, incorporating artificial intelligence-based image enhancement, object detection and segmentation to inspect 3D integration technologies utilized in QC ion trap devices. The workflow is suitable to gain sufficient statistical data and allows accelerated and accurate defect analysis. In particular, non-destructive SAM is applied to measure image data on wafer-level for a bonded-wafer containing ion trap recesses as well as for TSV connections used in QC. A wafer with defined test structures showing different length scales is utilized beforehand to benchmark different image enhancement models including classical as well as artificial intelligence (AI)-based super-resolution algorithms. The different model architectures are tested (1) on their ability to generalize on a large dataset and restrict unphysical hallucination but also (2) in terms of evaluation time and power consumption. Based on known metrics, the deep convolutional neural network with residual net, skip connection and a network in network (DCSCN) architecture showed its supremacy among classical and other ML-based image enhancement models. The workflow is enabled by passing the SAM image data to the mentioned super-resolution model, which increases image quality and, above all, enables further speed-ups in the SAM measurements. The artificially super-resolved images are then passed to an object-detection or segmentation algorithm to localize and classify defects in a specimen with TSV structures as well as analyze the adhesion quality of a bonded wafer specimen in a statistical manner. For the segmentation we utilize a ML-based U-Net architecture, whereas for the object detection, we introduce a fast YOLO approach, which speeds up the detection time by a factor of 60 in comparison to the state-of-the-art TSV object-detection methods. The introduced workflow yields overall a saving in time, including the experimental data generation, of roughly 4x and 6x for the TSV and delamination analysis, respectively. Notably, the workflow is not limited to SAM generated image data and demonstrates the capabilities of modern ML-powered methods in the wide field of semiconductor manufacturing.

## Results

### SAM measurement and data generation

**Fig. 1:**
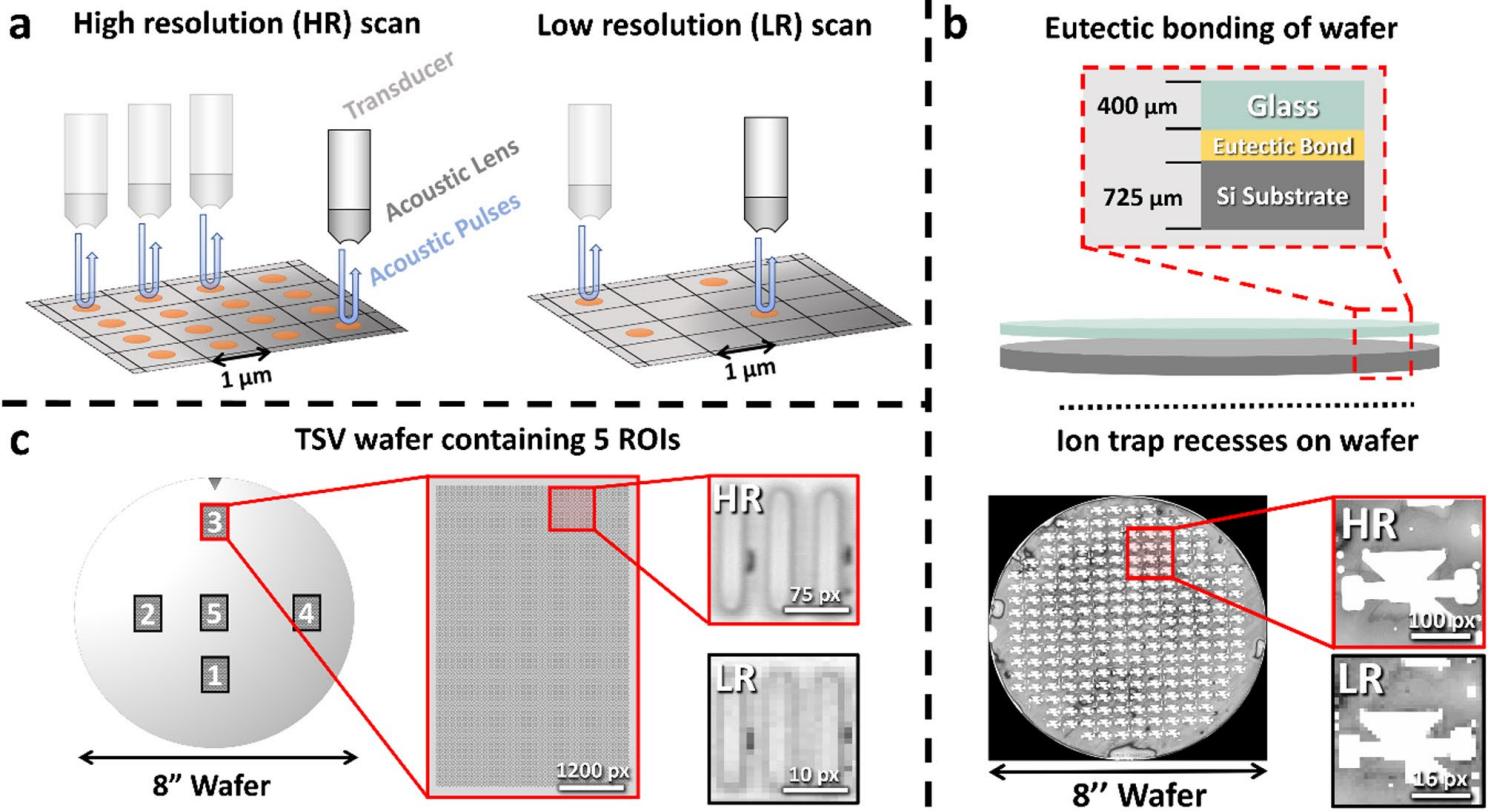
Scanning principle of a SAM and two different QC 3D integration technology specimens. (**a**) Scanning principle of SAM. To obtain a HR image, the transducer sends out and receives acoustic pulses at many scanning points. When using a low resolution, the transducer excites fewer pulses resulting in a shorter scanning time. (**b**) For the first specimen, a schematic of a bonded wafer is illustrated. A glass and unstructured silicon substrate, both fully metallized, are bonded together via eutectic bonding. A SAM C-scan image from the whole wafer containing the ion trap recesses (white grey values) is shown. Further grey values within the image can be associated with different qualities of the eutectic bond (light grey) and delamination (white and dark grey). Two magnified C-scan images for the same region of interest (ROI) are displayed on the right. They are indicated as HR and LR. (**c**) The second specimen shows a wafer with five TSV structures, each ROI exhibits 10,240 TSVs. The ROIs are highlighted with the numbers 1 to 5. A magnified image of ROI 3 is presented. A further zoom-in on the right highlights the TSV’s structure. HR and LR C-scan images are indicated. Winsam 8.24 software^61^ is employed for capturing and preprocessing the C-scan images.

We utilize C-Scan SAM to generate the experimental data. Figure 1a shows the basic working principle of a SAM device. The transducer produces acoustic pulses which are focused via an appropriate lens onto the sample. From the intensity and travel time of the reflected acoustic waves, information on structure and possible defects are extracted. Additionally, the scanning resolution of the SAM device can be lowered, resulting in a smaller resolution while speeding up the measurement time. Furthermore, the effective resolution depends on the frequency of the acoustic waves used^37^.

In this study, we exemplary investigate two specimens with two different 3D integrated technology-based building blocks on wafer level, crucial for the upscaling of trapped-ion QC devices. Figure 1b-c, illustrate the basic structure of the analyzed specimens. Further magnified C-scan images with different resolutions are provided. The first specimen, as shown in Fig. 1b, is fabricated by combining a fully metallized unstructured silicon as well as a glass substrate via eutectic bonding^26^ creating partly a MEMS based symmetrical 3D architecture providing more reliable trapping of the ions^60^, see Method section for further details. The ion trap recess is indicated on top of the wafer surface. We measure this wafer from the silicon side with two resolutions, namely with 300 μm/px and 50 μm/px. For this, we utilize a piezo-electric transducer with a focus length of 8 mm, finally permitting a center frequency of 209 MHz at the specimen. The focus for the C-scan SAM image is selected to be at the Si-eutectic interface at 5400 nanoseconds time-of-flight. Details with respect to time signal or A-scan are presented in Supplementary Note 1 and Supplementary Fig. 1.

The C-scan SAM image exhibits different grey values, which can be associated with the underlying different material phases and defect types originating from the eutectic bond between the wafers as well as delaminated areas. However, while the high-resolution (HR) 50 μm/px C-scan image displays sharp edges and good phase contrast, the low-resolution (LR) 300 μm/px image is pixelated and phases are harder to distinguish. This is especially problematic for resolution and contrast sensitive image analysis algorithms like object-detection and segmentation. In the utilized setting, the measurement of the 50 μm/px image takes around 6x longer than for the 300 μm/px image, due to its higher resolution. To leverage this problem and combine the high quality of the 50 μm/px image with the low scanning times of the 300 μm/px image, AI-based image enhancement will be used.

The second specimen, displayed in Fig. 1c, contains 10,240 TSVs per ROI. For a precise measurement of the TSV structure, which exhibits an extension of only about 8 pixels, we utilize a tone-burst setup, see Method section for further details. The center frequency of the transducer is 200 MHz, resulting in a frequency of about 205 MHz at the surface. The focus of the acoustic waves was selected to be at the surface of the wafer at around 1315 ns time-of-flight, the opening angle of the utilized lens in the transducer is 60°. For scanning the ROIs, a resolution of 2 μm/px was chosen. Using a resolution of 1 μm/px approximately quadruples the time needed, if all other scanning parameters stay the same. Hence, image enhancement is used to speed-up measurements by using a lower scanner resolution and simultaneously enhance the accuracy of object detection on those images. Further details regarding the specimens and setup are presented in the Method section and Supplementary Fig. 1.

### Workflow—From data acquisition over image enhancement to failure analysis

**Fig. 2:**
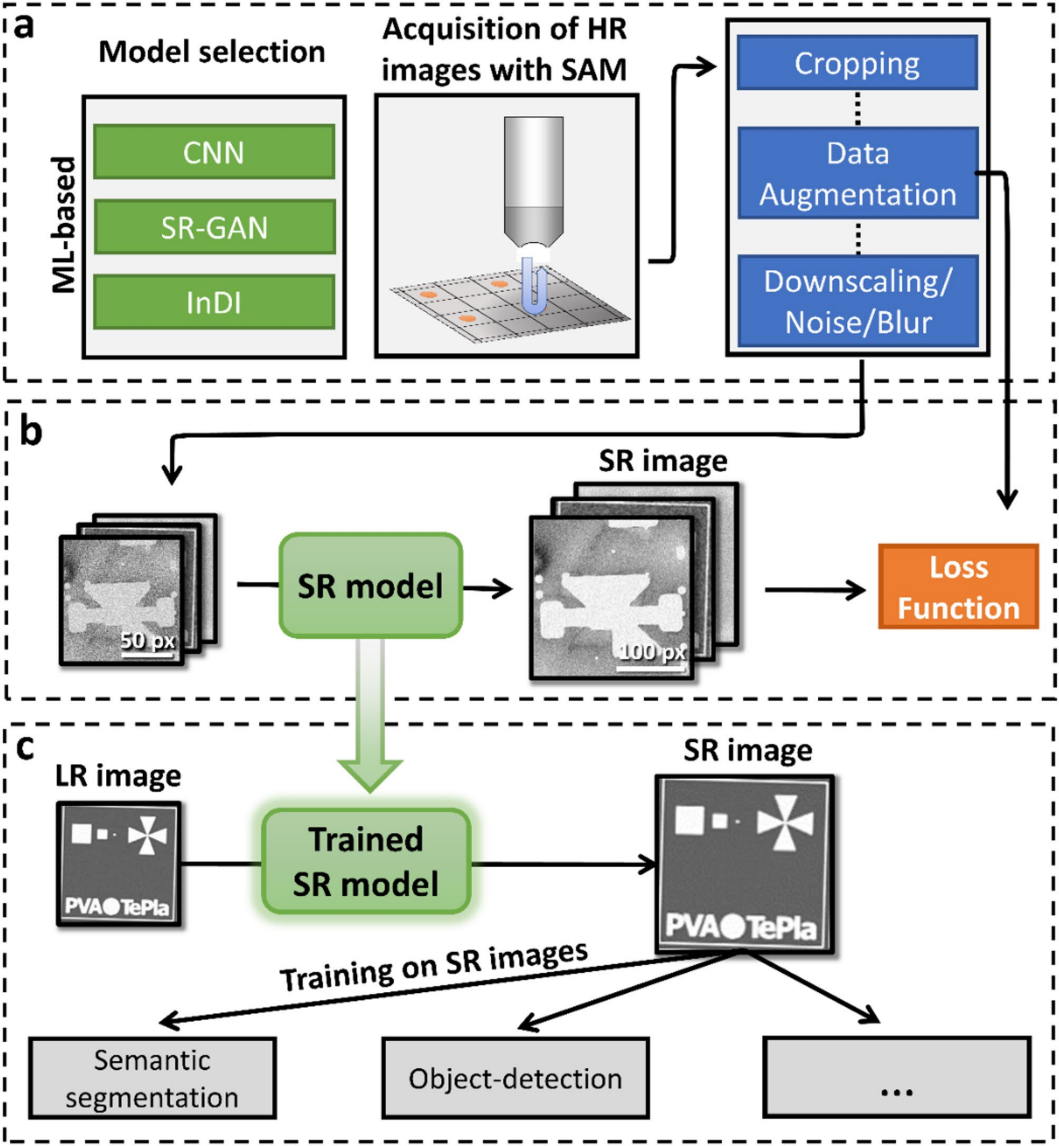
Overview of the super-resolution workflow. (**a**) The first step of the workflow consists out of model selection and data acquisition via SAM. The obtained C-scan images are cropped and augmented. To do self-supervised training, LR images are constructed by downscaling and altering the augmented HR images. (**b**) Training of the chosen model architecture utilizing the downscaled images. A predefined loss function guides the model training. (**c**) After training is complete, the model can be applied to enhance various other images. Further, the enhanced images can be used to improve the performance of subsequent resolution-sensitive algorithms like semantic segmentation or object-detection. Winsam 8.24 software^61^ is employed for capturing and preprocessing the C-scan images.

The overall workflow for super resolution (SR) and the downstream image analysis is shown in Fig. 2. It consists of three stages including model selection, data acquisition and preprocessing, self-supervised learning and application of the trained SR model to resolution sensitive failure-analysis tasks, see Fig. 2a-c, respectively.

As depicted in Fig. 2a, a SR model architecture and learning strategy has to be chosen. This can be a supervised CNN like DCSCN, a SR-GAN or an iterative algorithm like InDI. Additionally, high resolution image data has to be collected by using SAM. C-scan images are then preprocessed by cropping and augmentation.

Inspired by ideas of current self-supervised real-world SR approaches^50,62^ the augmented HR images are then downscaled by nearest-neighbors to produce the corresponding LR counterparts. Using a simple nearest-neighbors downscaling is justified by the fact that reducing the SAM scanning resolution is physically similar to deleting every second pixel in the image. To further ensure that the downscaled LR images looks realistic, multiplicative noise has to be added, since this is a common source of degradation in acoustic microscopy^39^. Lastly, we also employ Gaussian blurring and WebP compression to make the architecture more resilient to other degradation mechanisms. Multiplicative noise is applied with a probability of 30% and Gaussian blur as well as compression-noise is applied with a probability of 10% to every image. Details about training parameters and datasets used are available in the Methods section.

As can be seen in Fig. 2b, we use the final LR images as input to an exemplary SR model, which outputs images with higher-resolution. Image quality can now be measured in terms of a loss function to guide the training of the exemplary model. Nevertheless, this loss function can be chosen freely and the main problem comes down to avoiding regression-to-the mean, which causes blurry and less sharp image reconstructions^63^.

Depending on the quality and amount of training data, the SR model can now enhance various real-world images, see Fig. 2c. The models are trained on a wide variety of C-scans, enabling them to perform well on a large range of images including different resolutions and transducer types, see Methods section for more information. The enhanced images are then used for resolution-sensitive downstream tasks like semantic segmentation or object detection, often enabling improved performance due to higher image fidelity.

### Model selection and validation for image enhancement

**Fig. 3:**
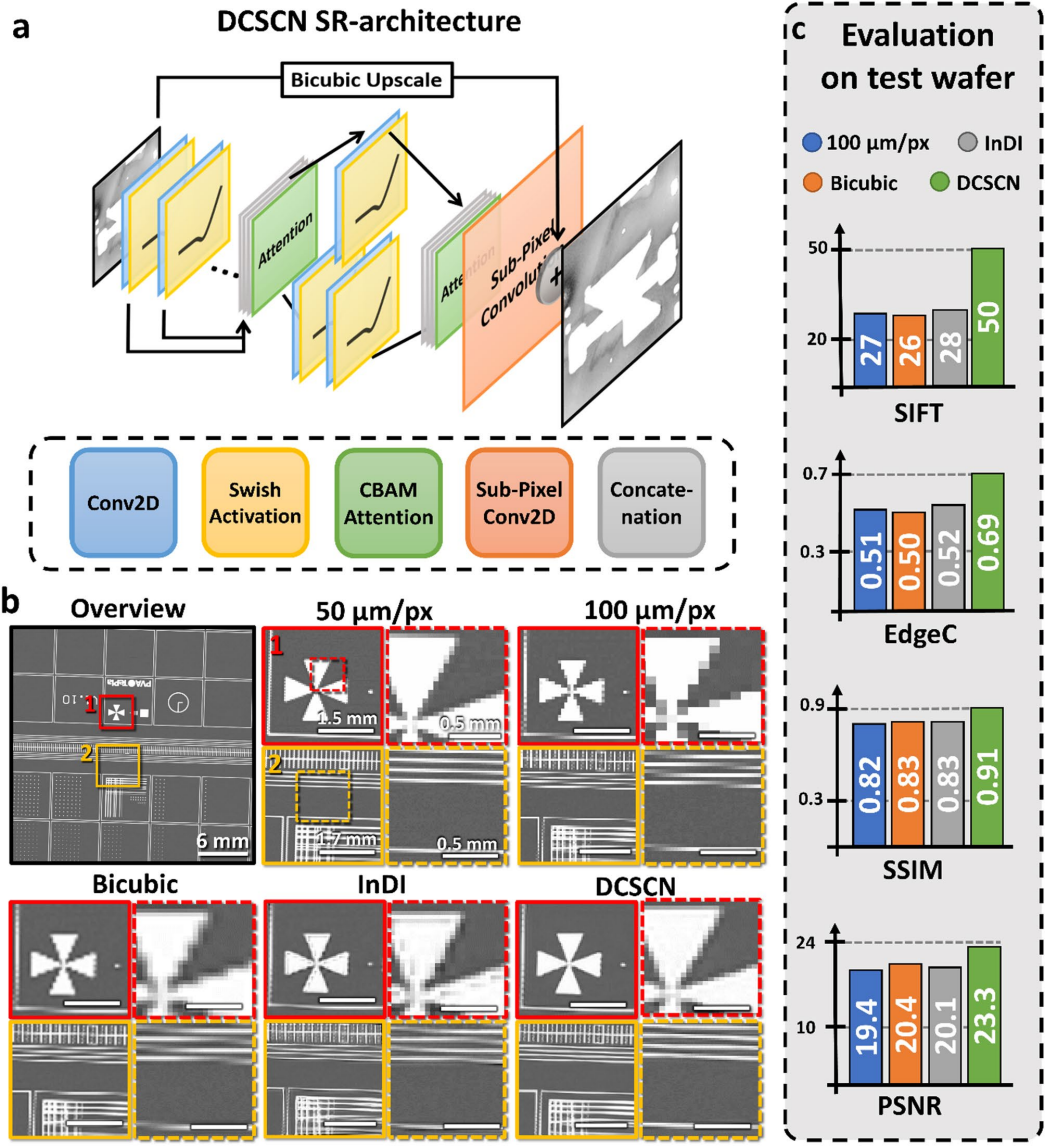
DCSCN model architecture and model evaluation of DCSCN and InDI SR. (**a**) Model architecture of CNN-based DCSCN SR model. The first block consists out of convolutional layers with 176, 160, 144, 128, 112, 96, 80, 64, 48 and 32 filters. The second block (reconstruction block) is split in two. It has convolutional layers with 32 and 32, 64 filters. The kernel size is 3 except for the first layers in the reconstruction block, where we use a kernel size of 1 for feature extraction. (**c**) Evaluation of SR on a test wafer. The upper left image shows an overview of the test structures. The colored images are zoomed in sections (ROI 1–2). ROI 1–2 are measured and displayed for different resolutions (100 μm/px and 50 μm/px). From the 100 μm/px we reconstruct a 50 μm/px image with bicubic interpolation, DCSCN and InDI. (**d**) PSNR, SSIM, EdgeC and the number of matched features found via a SIFT algorithm are listed as bar graphs. They show a clear advantage of the DCSCN approach compared to classical bicubic upscaling, but close to no improvement when using InDI. Winsam 8.24 software^61^ is employed for capturing and preprocessing the C-scan images.

For image enhancement we train various modern ML-based SR architectures and compare them to classical methods, see also Table 1. The developed image enhancement shall foster to eliminate time limitations fetched by the experimental HR scans by doubling the resolution after measurement, as shown in Fig. 3. Most importantly, the SR approach should also generalize to various scanning resolutions and transducer types. To achieve this, self-supervised model training is implemented, allowing to train on much larger dataset and improving generalizability. Moreover, the ML-models are discussed not only based on the performance gained by known metrics but also by their evaluation time per image as well as energy consumption.

One can quantify the reconstruction quality of different models by calculating common metrics like the peak signal-to-noise-ratio (PSNR) and structural similarity index measure (SSIM)^63,64^. Both allow a comparison to other models found in literature. However, these two metrics are sensitive to small image transformations and do not capture important image characteristics like sharp edges^44,63,65^. Therefore, they do not present useful objectives for measuring overall real-world performance, and we aim to introduce two new metrics which try to capture more of the physical information. The first metric is called edge correlation index (EdgeC). It uses a canny edge detection algorithm to detect edges and calculates the correlation function between the detected edges in the HR and reconstructed image. Possible values of EdgeC range from + 1 to -1, corresponding to perfect correlation or anti-correlation. Furthermore, we introduce a metric based on the scale-invariant feature transform (SIFT) algorithm^66,67^. SIFT is a popular method to find congruent points in two images. We can employ this algorithm and count how many congruent points SIFT detects between both images. The higher the count, the better the reconstruction. More details about these metrics are presented in the Supplementary Note 2, Supplementary Fig. 2 and Supplementary Table 1.

Table 1 indicates the performance of bicubic and nearest neighbor upscaling in terms of the self-supervised regime, where the LR images are produced by artificially downscaling HR C-scan images. It is obvious that bicubic and nearest-neighbor upscaling perform poorly in terms of the introduced metrics. Nevertheless, when using AI-based models, there are several possibilities for selecting the loss function and training, leading to better reconstruction quality.

One common approach to achieve high-quality outputs is by the use of GANs. To test the capabilities of GAN models for the SR tasks, we implement a SR-GAN^41^. The generator has the same architecture as displayed in Fig. 3a and is trained with a combination of perceptual loss and adversarial loss, the latter representing the feedback from the discriminator. The discriminator itself is trained using a relativistic average loss^68^. As shown in Table 1 this SR-GAN approach shows better performance than classical models across all metrics.

Another way to produce high-quality images is by using an iterative algorithm. For this, we implement the recent inversion by direct iteration (InDI) diffusion-like algorithm, which uses a LR image and gradually increases its quality step by step^43^. As seen in Table 1 InDI performs good for artificially downscaled image data. However, InDI shows issues for the measured low resolution SAM image data, see Fig. 3b-c. There, real measurements of a wafer with test-structures, obtained with 50 μm/px and 100 μm/px resolution directly on the SAM, are shown. It is noticeable that the InDI algorithm is not able to reconstruct the straight lines in ROI-2 from the 100 μm/px image. Additionally, the InDI model hallucinates structures which are not there in the real HR image, as can be seen close to the edges of the cross in ROI-1. This further underscores the importance for real-world evaluations, especially for highly generative and iterative models like InDI. In fact, the problem of hallucinations in highly generative models is gaining increasing attention in the last years^51,52^. Similar comparisons on real-world data using the SR-GAN model can be found in Supplementary Note 3 and Supplementary Fig. 3.

Perceptual loss functions^46^ are another common way to produce high-quality outputs in SR tasks. We chose to implement such a perceptual loss function, employing a feature extraction neural network for extracting important features and structure from the image. The mean-averaged-error (MAE) is then calculated between those extracted features, see Method section for further details. With this loss function, the state-of-the-art SRResNet (Super Resolution Residual Network)^40^ is implemented, which gives results close to SR-GAN and InDI in Table 1. However, when applied to real-world data, the SRResNet performs only slightly better than bicubic upscaling, as demonstrated in Supplementary Fig. 3.

Last but not least, we also implement a more complex fully convolutional neural network based on an adapted DCSCN architecture^42^ trained with the same perceptual loss as SRResNet. The DCSCN architecture is exemplarily shown in Fig. 3b. Surprisingly, this model shows the best performance across nearly all metrics presented in Table 1, even outperforming the generative models like SR-GAN and InDI, as well as the SRResNet. Furthermore, DCSCN is superior to other methods under real-world applications, as displayed in Fig. 3c and Supplementary Fig. 3. 

Table 1 also includes data for the evaluation time and energy consumption during training. To train the diffusion-like InDI and generative SR-GAN models, more powerful hardware has to be used, which also increases the energy consumption and environmental footprint by a factor of around two. Due to its larger parameter size and iterative approach, InDI also takes roughly one order of magnitude longer to reconstruct images. In fact, DCSCN seems to present the best balance between image quality, evaluation time and power consumption.

Detailed information about the SR-GAN, InDI, SRResNet and DCSCN architectures and training can be found in the Methods section, Supplementary Note 4 and Supplementary Fig. 4.

**Table 1:** Evaluation of the SR models on randomized SAM images.

	Model	PSNR (↑)	SSIM (↑)	Edge-correlation (↑)	SIFT (↑)	Evaluation time per image (↓) (s)	Energy consumption - training (↓)
Classical	Nearest-Neighbors	27.8	0.86	0.47	16.9	< 0.01	0 kWh
	Bicubic	29.8	0.90	0.47	20.0	< 0.01	0 kWh
ML-based	SR-GAN	34.3	0.91	0.77	32.9	0.10	~ 15.1 kWh
	InDI	34.4	0.93	0.74	32.2	10.0	~ 18.0 kWh
	SRResNet	34.0	0.87	0.79	34.2	0.05	~ 3.9 kWh
	DCSCN	35.1	0.92	0.79	34.5	0.10	~ 7.9 kWh

For calculating metrics 1000, 256 × 256 pixels C-scan images are first downscaled to 128 × 128 pixels by deleting every second pixel. Then, the LR image is passed to different SR models to be reconstructed. Different metrics are evaluated by comparing the original HR image with the reconstructions. Values obtained by nearest-neighbor and bicubic upscaling are provided as reference. Additionally, we show the evaluation time per image, tested for a batch of 10 images with 256 × 256 pixels on a Nvidia RTX A5000 with 24Gb VRAM, and approximate energy consumption in kWh used for training. See the methods section for more information on the hardware used for training.

### Failure-analysis of a bonded ion trap wafer

**Fig. 4:**
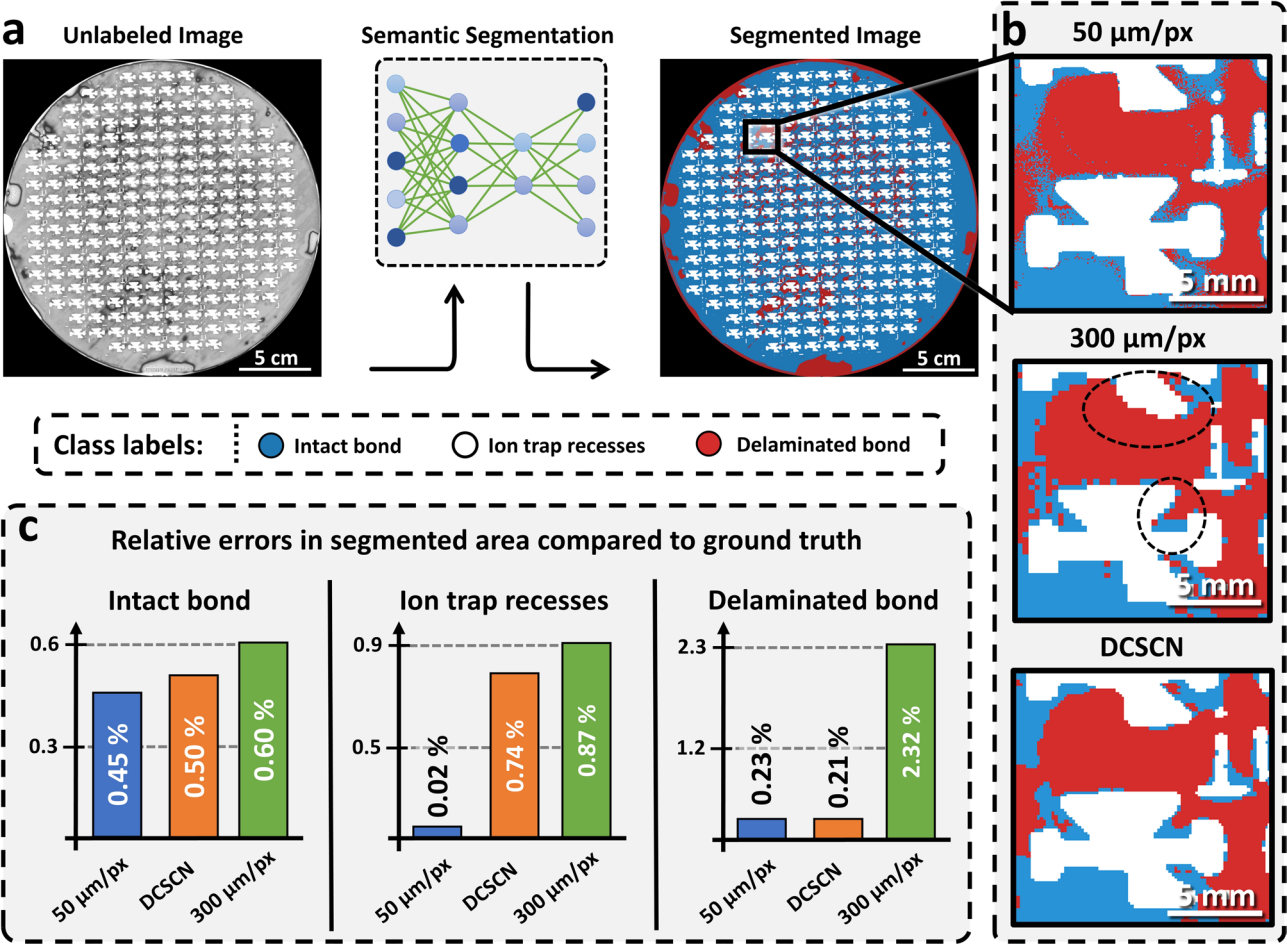
Bond quality evaluation of an eutectically bonded ion trap wafer. (**a**) Demonstration of ML-based segmentation using a residual attention U-Net. Three classes are distinguished: ion-trap recesses (white), intact eutectic bond (blue) and delaminated/incomplete bond (red). (**b**) Magnified area of the segmented wafer with a resolution of 50 μm/px, 300 μm/px and an image illustrating 300 μm/px with the applied DCSCN model, from top to bottom. Significant deviations of the 300 μm/px image from the 50 μm/px image are indicated by dashed black circles. Clearly the DCSCN and 50 μm/px images indicate higher similarity. (**c**) Relative errors in various segmented phases when compared to the manually labeled ground truth for the 50 μm/px, 300 μm/px and DCSCN-enhanced image. Winsam 8.24 software^61^ is employed for capturing and preprocessing the C-scan images.

To test the capabilities of SR in industrial applications, we apply the selected CNN-based DCSCN model to the eutectically bonded wafer specimen displayed in Figs. 1b and 4a. The main goal is to show how SR can decrease the time for large-scale SAM measurements and improve the accuracy of subsequent segmentation-based failure analysis.

We again note that C-scan images of the wafer with 50 μm/px and 300 μm/px resolution are available, whereas the 300 μm/px resolution is close to the resolution limit for detecting small features. Different structures, material phases and defect types are visible in the C-scan image, see also Methods section. To quantify the bond quality of the wafer, the scanned images are segmented into 3 distinct regions and the corresponding areas are evaluated, see Fig. 4a. In particular, we distinguish between ion-trap recesses (white), intact eutectic bond (blue) and delaminated eutectic bond (red). For segmentation, three separate state-of-the-art residual attention U-Net^69^ models, for the three different resolutions (50 μm/px, 300 μm/px and DCSCN enhanced), are trained and employed. More information on the training for the segmentation model is provided in the Methods section.

In Fig. 4b a cutout of the segmented C-scans for a resolution of 50 μm/px, 300 μm/px as well as the DCSCN-enhance image are presented. Clearly, deviations between all images can be depicted, especially between the 300 μm/px and 50 μm/px images. In hard to segment areas, like for the upper ion-trap recess in Fig. 4b, the U-Net segmentation model trained on the 300 μm/px image struggles to detect the whole ion-trap structure. In comparison, even though the DCSCN enhanced image seems to be smoothened and loses some details in comparison to the 50 μm/px image, it is obvious that there is a better qualitative correspondence and all key features are properly segmented in this case.

Figure 4c provides a quantitative comparison of the relative errors in segmented areas between the 50 μm/px, 300 μm/px, DCSCN-enhanced image and a manually labeled ground truth. When applying the DCSCN model to the 300 μm/px image, a decrease of the relative error by at least 10% or more can be established. There are three main reasons for the observed improvement. First, the LR 300 μm/px image is pixelated, leading to lower-details in fine structure and, therefore, a different area of the phases. Second, manual labeling of the LR image for subsequent training of the U-Net is more difficult due to the decreased edge-contrast, making it harder to accurately train the model. Third, when a model is trained with LR data, it has a lower amount of pixel-data to be trained with. For example, the 300 μm/px image has 36 times less pixels then the 50 μm/px image, decreasing model performance and generalizability. All these three factors can be improved by applying super-resolution before manual labeling and model training. Also, according to this reasoning, the provided findings are general and carry over to different model architectures as presented in the Supplementary Note 5 and Supplementary Table 2.

### Fast object detection and super-resolution for through-silicon-vias (TSVs)

For the failure analysis of thousands of TSVs, we localize and classify every individual TSV on the wafer, see also Fig. 1c. We implement and compare different ML-based object detection algorithms including YOLOv2^70^ and YOLOv12^71^. YOLO is a so-called one-shot method, since it localizes and classifies all objects in an image within one evaluation of the neural network. This makes the method very time efficient, especially for large images.

**Fig. 5:**
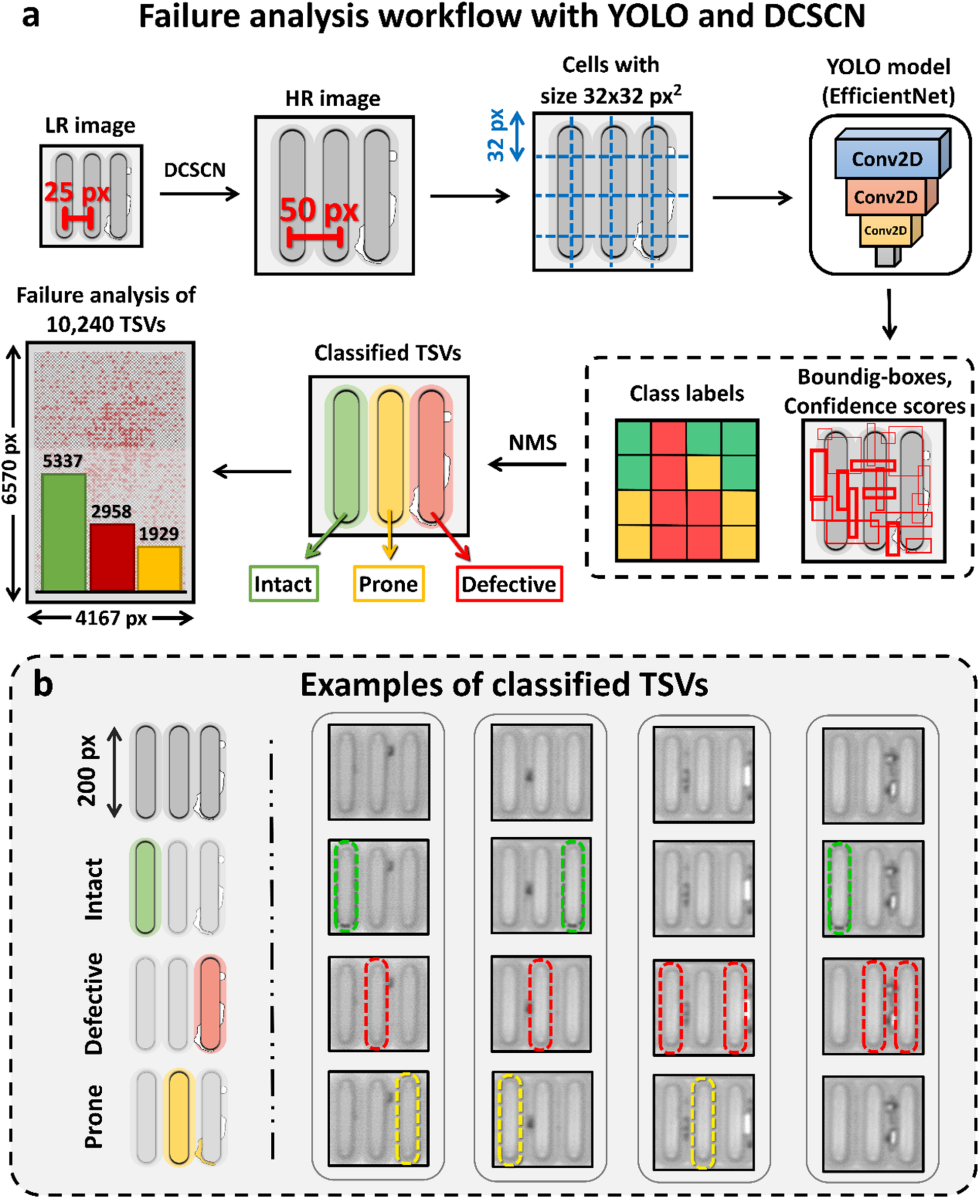
Workflow to enable YOLO object detection with SR and definition of defect labeling. (**a**) YOLOv2 object detection pipeline. We start by increasing the resolution of the LR scanned image by 2 times, to increase the distance between adjacent TSVs. After that, the HR image is divided into cells of 32 × 32 pixels and evaluated by the YOLO model. The YOLO model utilizes an EfficientNetV2-B0 backbone. The outputs of the model are class labels, bounding boxes and confidence scores for every grid cell. In a last step, NMS is used to filter out intersecting boxes with low confidence. This algorithm can now be used to carry out large scale failure analysis as shown for a ROI containing 10,240 TSVs. (**b**) TSV classification and measurements. We sort TSVs in three classes: Intact TSVs (green), defective TSVs (red) and TSVs which are prone to be impacted in functionality due to nearby defects (yellow). Winsam 8.24 software^61^ is employed for capturing and preprocessing the C-scan images.

Figure 5a shows the basic steps of the failure analysis workflow. The workflow starts by applying SR to the input image to double its size, then dividing it into a grid of cells. For YOLOv2, cells with a size of 32 × 32 pixels are usually used. For every grid cell, a neural-network then predicts three values namely, (1) a confidence score, which measures the probability of an object being present in the cell, (2) the bounding box coordinates of the object and (3) its class labels. Finally, non-maximum suppression (NMS) is used to filter out overlapping boxes with low confidence score and a statistical evaluation can be carried out.

In Fig. 5b three quality classes for the TSVs are defined. The first class contains fully intact TSVs without any sort of defect or other imperfection. The second class defines defective TSVs. This category is characterized by black or white imperfections around the edges of the TSV. The third class covers TSVs where a failure cannot be ruled out completely, e.g. they are prone to be impacted in functionality. These TSVs have a defect close to their boundary, however, the defect does not touch the TSV itself.

As a matter of fact, detecting small objects, like the TSVs shown in Fig. 5, displays critical problem for every object-detection algorithm^59^. Table 2 shows that all tested object detection algorithms show increased performance when trained and evaluated on the DCSCN-enhanced SR images and perform worse when trained and evaluated on the original LR images. The YOLOv2 algorithm is not even able to converge to a proper state, since its cell size is 32 × 32 pixels, limiting the model to only distinguish between objects with a minimum distance of 32 pixels. However, the TSVs illustrated in the C-scan image data have a distance of 25 px, therefore, being too close for YOLOv2 to distinguish. In contrast to this, YOLOv12, which uses multi-scale training and smaller cell sizes, is still able to localize and classify TSVs on the LR images, however, with reduced accuracy. In fact, detection accuracy for both, YOLOv2 and YOLOv12, reaches 99.8% on the SR images. This means, that only 2 out of one thousand TSVs are not detected.

The classification accuracy for sorting the TSVs into the three classes defined in Fig. 5b is evaluated to be around 96% for all models trained on the SR images, and thus close to the capabilities of the approach presented in^34^, however with higher time efficiency. For example, the evaluation of 10,240 TSVs takes only around 8 s for YOLOv2. To further emphasize the time-efficiency of the YOLO model, we compare it to the recently introduced end-to-end sliding window approach^34^ by applying it to the data provided in^34^, see Supplementary Note 6 and Supplementary Fig. 5. Note that the presented YOLOv2-based model architecture outperforms, in terms of time, the mentioned end-to-end sliding window approach^34^ by a factor of 60.

Table 2 also includes a transformer based Real-Time Detection Transformer (RT-DETR) object-detection model^56^. Even though this model performs good for the SR images, it underperforms in terms of detection accuracy compared to YOLOv12 on the original LR images. Also, since RT-DETR is transformer-based, model inference can only be applied on images of the same size as the training images. This is a drastic practical shortcoming since object-detection is often trained on small image crops and then applied to larger images. See the Methods section for more details.

**Table 2:** Accuracy of different object detection models trained and evaluated on SR images and LR images of half size.

LR/SR	Classification Accuracy	Detection Accuracy	Model Parameters (Million)
YOLOv2			
Low-Resolution	Training not converged		5.90
Super-Resolution	95.7%	99.8%	5.90
YOLOv12s			
Low-Resolution	93.8%	98.2%	9.25
Super-Resolution	96.9%	99.8%	9.25
RT-DETER-l			
Low-Resolution	94.8%	84.6%	32.8
Super-Resolution	96.6%	96.3%	32.8

YOLOv2 and YOLOv12 are CNN based one-shot models. Since YOLOv2 is limited by a cell size of 32 Px, it does not converge to a proper state for the LR images since TSVs are only 25 Px apart. RT-DETR is a transformer-based model. Even though performance is high for the SR images, detection performance drops fast at lower resolution. Additionally, RT-DETR has the highest number of parameters with the highest computational cost.

## Conclusion

Ultimately, for the establishment of QC into the society and its industrialization, qubit up-scaling is crucial. The implementation of 3D integration to QC provides a highly beneficial platform and provides possibilities in this context. This also sets new challenges for failure analysis.

In this paper, we demonstrate how to leverage ML-based SR techniques to enhance SAM imaging for failure analysis and quality assessment in 3D integration technology utilized for advanced QC devices. In particular, we tested different modern model designs to enable SR, including GAN and diffusion-like approaches. Among these architectures, the DCSCN model showed the most consistent performance across all tested metrics, providing superior results in PSNR, canny edge-correlation and SIFT-based evaluations. Further, the DCSCN model is able to balance time-efficiency and output quality, featured by a decreased power consumption during training and enabling a reduced environmental footprint. Beyond that, the tested GAN and diffusion-like models are prone to unphysical hallucinations, which are unwanted for real-world applications.

The findings also highlight the ability of a ML-SR approach to reduce scanning time while preserving essential defect-related information. For example, integrating a SR model into the presented workflow enables reliable segmentation and quality-assessment of wafer bonding, even when a low-resolution C-scan image is used. This offers enhanced possibilities for high-throughput quality assessment in industrial applications. Employing SR and the discussed measurement setup, scanning times can be reduced by a factor of up to 6x. Note, that scanning times may vary slightly due to different measurement setups and scanning parameters.

Notably, SR also makes it possible to apply fast object detection algorithms like YOLO. In particular YOLO is used for the detection of 10,240 TSVs, enabling advanced statistical analysis. Due to the small size of the TSVs, common object detections approaches fail in detecting and localizing large amounts of TSVs with high accuracy. This can, however, be solved with SR. When applying SR, a detection and classification accuracy of up to 99.8% and 96.9%, respectively, can be reached. This leverages the presented approach to overcome problems posed by small TSV sizes and associated long scanning times. In fact, SR enables a reduction in scanning time by a factor of about 4x in this case, assuming that the scanning time increases quadratically with resolution. Furthermore, the employed YOLO architecture reduces the time for carrying out object detection by a factor of 60x when compared to recently introduced approaches^34^.

In conclusion, ML-SR approaches like the DCSCN offer a scalable and efficient solution for high-throughput defect characterization in quantum-circuit- and semiconductor-manufacturing. The results underscore how ML-based image processing can be utilized to address challenges related to resolution, time efficiency and accuracy in industrial applications.

## Methods

### Ion trap specimens

The first specimen shown in Fig. 1b indicates the bonded sample resembling the bottom two layers of a MEMS based symmetrical 3D ion trap architecture^58^. The bonded specimen consists of a 725 μm-thick silicon substrate as the bottom wafer with a SiO_2_ passivation layer on which a 600 nm thick eutectic component 1 is sputter deposited. The top wafer is made of 400 μm-thick borosilicate glass featuring recess structures through the whole wafer, which are created by wet-chemical etching. A 550 nm thick eutectic component 2 is deposited onto the glass wafer by evaporation. Eutectic component 1 and eutectic component 2 are unstructured. The silicon and borosilicate glass wafers are bonded using eutectic bonding.

The second specimen as shown in Fig. 1c displays a wafer with TSV structures. It features five 0.8 cm x 1.28 cm ROIs containing 10,240 TSVs in a 500 μm-thick silicon substrate. Fabrication is based on a 725 μm-thick silicon on insulator substrate (SOI) with a bottom 500 μm-thick silicon layer, a 1 μm-thick buried silicon oxide (BOX), and a 225 μm thick top active layer (TAL) containing alternating silicon and silicon oxide layers. The TSV recesses are created by deep reactive-ion etching of the 500 μm-thick silicon. Filling of the recesses is done by low pressure chemical vapor deposition of doped silicon. Using the BOX as a stop layer, the top silicon layer is removed by mechanical grinding and subsequent chemical-mechanical polishing (CMP). A subsequent CMP step is employed to remove residual silicon layers on the back side opening up the TSVs. Therefore, a silicon oxide layer is deposited as a passivation layer on the back side using plasma-enhanced chemical vapor deposition.

The bonded specimen fabrication was carried out in industrial cleanroom facilities at Infineon Technologies in Villach, Austria. For the TSV specimen the facilities at Infineon Technologies in Regensburg, Germany was utilized.

### Detailed measurement setup

We use a SAM 302 HD^2^ provided by PVA TePla. For all measurements a Winsam 8.24 software^61^ is employed for capturing and postprocessing the C-scan images. HQ setting was enabled for all measurements. The ADC employed, has a sampling rate of 5 GSamples/s.

TSV measurements presented in the paper are obtained using a tone-burst setup utilizing a transducer with a peak frequency of 200 MHz and opening angle of 60°. The measured peak frequency at the sample is 205 MHz. The surface of the wafer was used as focus point having a time-of-flight distance on 5400 ns.

The second specimen, a bonded wafer, is measured using two scanning resolutions (300 μm/px and 50 μm/px) utilizing a transducer of VHF+ (very-high-frequency plus) type with 8 mm focus length, a nominal frequency of 160 MHz and a center frequency of 209 Mhz reflected by the sample. The Si-eutectic interface is used as focus point for the acoustic waves having a time-of-flight distance of 1315 ns.

### Details of the model architectures for super-resolution

In the presented paper, four different models for the self-supervised super-resolution approach are tested: Two purely CNN based models called (1) DCSCN (Deep CNN with Residual Net, Skip Connection and Network in Network) and (2) SRResNet (Super-Resolution Residual Network), (3) one GAN based model (SR-GAN), and (4) a diffusion-like architecture called InDI (Inversion by Direct Iteration).

The architecture of the DCSCN model is displayed in Fig. 1b and is similar to the architecture introduced in the original DCSCN paper^42^. The only difference is the use of swift activation to prevent the dying neuron problem, the introduction of CBAM attention blocks^72^ before each bottleneck layer, and a slightly adapted filter count starting with 177 filters in the first layer and reducing the filter count in steps of 16 to a minimum of 32. For the final upscaling, a sub-pixel convolution is used. The kernel size is 3 in every layer except the ones directly after the first concatenation. There, we choose a kernel size of 1 for feature extraction.

The SRResNet architecture is taken directly from the original paper^40^ only changing the activation function from prelu to swift. We used 24 residual blocks without batch normalization.

For the SR-GAN we adjust the architecture presented in the ESR-GAN paper^41^. The generator is changed from SRResNet to DCSCN, because DCSCN performs better. The discriminator is displayed in Supplementary Fig. 4 and is employing swish activation in combination with batch normalization. Strided convolutions are used for down sampling.

The InDI model is based on the original DDPM U-Net architecture^45^. Again, the only difference being the use of swish activation in contrast to relu activation. For evaluation, 10 iterations were used. When using more than 10 iterations the InDI approach produced images with constantly degrading quality.

### Detailed training settings for the super-resolution models

Except for the training of the SR-GAN, all presented SR models employ a one-cycle schedule^73^ with a cosine decay and AdaBelief^74^ optimizer. One-cycle schedule and AdaBelief have been shown to improve convergence speed compared to other training schedules and optimizers. Furthermore, the one-cycle schedule, due to its increasing learning rate, acts as a regularizer in the middle of the training. For SR-GAN we use an exponentially decaying learning rate with adam optimizer to prevent unstable training.

The training set consist of around 300 large C-scan images from the SAM. Additionally, we add the DF2K and OST datasets to give the model the ability to generalize to a broader range of datasets^75–77^. This is justified by the fact that super-resolution models mainly learn structures in images, and typical structures like circles, lines and others are present in all mentioned datasets.

Since the datasets contains C-scan images with varying sizes, we split all images into patches of 256 × 256 pixels. Random rotation, random flipping as well as random pixel inversion are applied to the HR and LR images for augmentation purposes. In contrast to this, the image modifications for self-supervised training (multiplicative noise, gaussian blur and image compression) are only applied to the low-resolution images.

The loss function used for training DCSCN and SRResNet is a perceptual loss function. For this loss function a VGG19 feature extraction network, pretrained on ImageNet, is employed. We use the activations of the second convolution in the second block ($\:{\phi\:}^{\text{2,2}}$) as features and calculate the MSE loss between the features of high-resolution and reconstructed image. Also, we add a small MAE and TV loss to reduce unwanted high-frequency artefact. The detailed loss is

$$\:{L}_{p}=MSE\left({\phi\:}_{HR}^{\text{2,2}},{\phi\:}_{LR}^{\text{2,2}}\right)+0.1*MAE(HR,LR)+5{*10}^{-5}*TV.$$

Both models are trained for 700k steps with a batch size of 16 on a NVIDIA Quadro P4000 GPU with 8 Gb of V-RAM and Intel Xeon Silver 4108 CPU with 64 Gb of RAM.

For the GAN training, three losses with relative sizes of 1/20, 1/50 and 1 are utilized: The perceptual loss mentioned above, an adversarial loss and a discriminator loss. The perceptual loss makes sure that the generated images stay close to the original HR images, whereas the adversarial loss gives the generator feedback about how realistic the generated images look like. Both, discriminator and adversarial losses are based on a relativistic average loss, which has been shown to reduce instabilities in GAN training^68^. To further increase training stability, we train the generator four steps, for every step of the discriminator. The reason behind this is that it is much harder to paint a Picasso, then to tell if a painting is a fake Picasso. Therefore, the training of the generator should be focused on. Lastly, the starting weights of the generator are set to the $\:{L}_{p}\:$pretrained DCSCN weights and training was done for 29k steps with 32 batch size on a NVIDIA A40 GPU with 48 Gb V-RAM and AMD EPYC 7513 32-Core CPU with 1024 Gb RAM.

For training the InDI model, a simple MSE loss is employed, similar to what was shown by the original authors^43^. The training was done for 117k steps with a batch size of 64 on a NVIDIA A40 GPU with 48 Gb V-RAM and AMD EPYC 7513 32-Core CPU with 1024 Gb RAM.

All preprocessing, training and the whole architecture design was carried out in python TensorFlow 2.10 and Keras 2.15. The power consumption depicted in Table 1 is calculated using the information form the hardware manufacturer and observed training time assuming an average utilization of 80% for GPU and 30% for the CPU.

### Training and architecture details for YOLO and RT-DETR object detection

The mentioned YOLOv2 object detection model is based on an EfficientNetV2-B0 backbone^78^. The last part of the network consists out of a convolutional and a reshape layer to change the EfficientNet output to the required YOLO format. Here, the output is a matrix where every entry corresponds to one gird cell of the image. The entries themselves are vectors of length (4 + 1 + number of classes). The first four numbers specify the x and y position of the bounding box relative to the grid cell as well as the width and height of the bounding box. The fifth entry in the vector is the confidence score, telling us how likely it is that an object is contained in the image.

In fact, since we employ a YOLOv2 algorithm, the model output is not one matrix but several ones, each matrix corresponding to one anchor box. Anchor boxes are predefined bounding boxes, which give the model a starting point for finding the right size of objects. The optimal shape of these anchor boxes is usually found via a K-Nearest Neighbor algorithm. It turns out that two anchor boxes with the size of the TSVs, oriented vertically and horizontally, are obtained. The model outputs two matrices corresponding to the two anchor boxes. The one with the higher confidence score is then chosen as the final output.

YOLOv12s and RT-DETR-l are implemented using the standard implementation provided by Ultralytics without changing any hyperparameters.

For training, 832 × 832 image patches are used. These are obtained by splitting larger images into tiles. Since YOLO models are fully convolutional, they do not depend on the image size and generalize to other image-sizes without problems. This is not the case for the transformer-based RT-DETR model, since it implements a fixed positional encoding. The labeling is carried out with the labelme python implementation^79^. In total, we use a training set of 200 images containing TSVs from one die. For training, we employ a CosineRestart schedule till convergence (36k steps for YOLOv2) and a batch size of 8. The accuracy results stated in the main text were obtained by using ROI 1 for training and ROI 3 for testing. YOLO based models were applied to the whole die at once, whereas for RT-DETR the image of the die was split into patches of 832 × 832 pixels.

All preprocessing, training and the whole architecture design for YOLO was carried out in python TensorFlow 2.10 on a NVIDIA RTX A5000 GPU with 24 Gb V-RAM and Intel Xeon w5-3433 CPU with 512 Gb RAM.

### Semantic segmentation data, models and training parameters

All images are labeled with dragonfly and split in two halves, one for training and one for testing. Due to the sparse amount of training data, augmentation for training is employed. We use random crops to ¼ of the shortest image size, flipping, rotation, brightness changes and random-grid shuffle. Out of every training image we obtain 1200 augmented images.

The attention residual U-Net^69^ mentioned in the results section is implemented. The model is trained for 70 epochs with early-stopping, an exponential decaying learning rate (lr_strart = 0.007, decay = 0.7/epoch), adam optimizer, batch size of 8 and sparse-categorical-crossentropy loss. The U-Net model has a depth of 4 and a starting filter-count of 8. The filter count doubles after every down sampling layer. Residual blocks are implemented according to^80^ but using swish activation.

All preprocessing, training and the whole architecture design for YOLO is carried out in Python TensorFlow 2.10 on a NVIDIA RTX A5000 GPU with 24 Gb V-RAM and Intel Xeon w5-3433 CPU with 512 Gb RAM.

## Electronic supplementary material

Below is the link to the electronic supplementary material.

Supplementary Material 1

## Data Availability

Data is provided within the manuscript or supplementary information files. Further data if necessary is available from the corresponding author upon request.
