# Supplementary material for: AI image enhancement for failure analysis in 3D quantum information technology
Source: Sci Rep. 2025 Jul 5;15:24078. doi: 10.1038/s41598-025-08308-4 (PMC12228736; doi:10.1038/s41598-025-08308-4)
Supplement: Supplementary file 1 — Supplementary Material 1 [file 41598_2025_8308_MOESM1_ESM.pdf]

Supplementary File:

AI image enhancement for failure analysis in 3D quantum information technology

Raphael Wilhelmer[1], Fabian Laurent[2], Tatjana Djuric-Rissner[3], Max Glantschnig[2], Johann Strasser[4], Stefan Weinberger[4], Tobias Herrmann[4], Clemens Rössler[2], Peter Czurratis[3] & Roland Brunner[1,*]

[1] Materials Center Leoben Forschung GmbH, Leoben, Austria

[2] Infineon Technologies AG, Villach, Austria

[3] PVA TePla Analytical Systems GmbH, Westhausen, Germany

[4] Infineon Technologies AG, Regensburg, Germany

*corresponding author: roland.brunner@mcl.at

SUPPLEMENTARY NOTE 1: A-Scan images of SAM measurements

In Supplementary Figure 1 representative A-scans for the two different wafers investigated in the main text (bonded and TSV wafer) are shown. These images show the position of the surface trigger and gates. For the tone-burst measurement of the TSVs a long data gate without a surface trigger was applied.

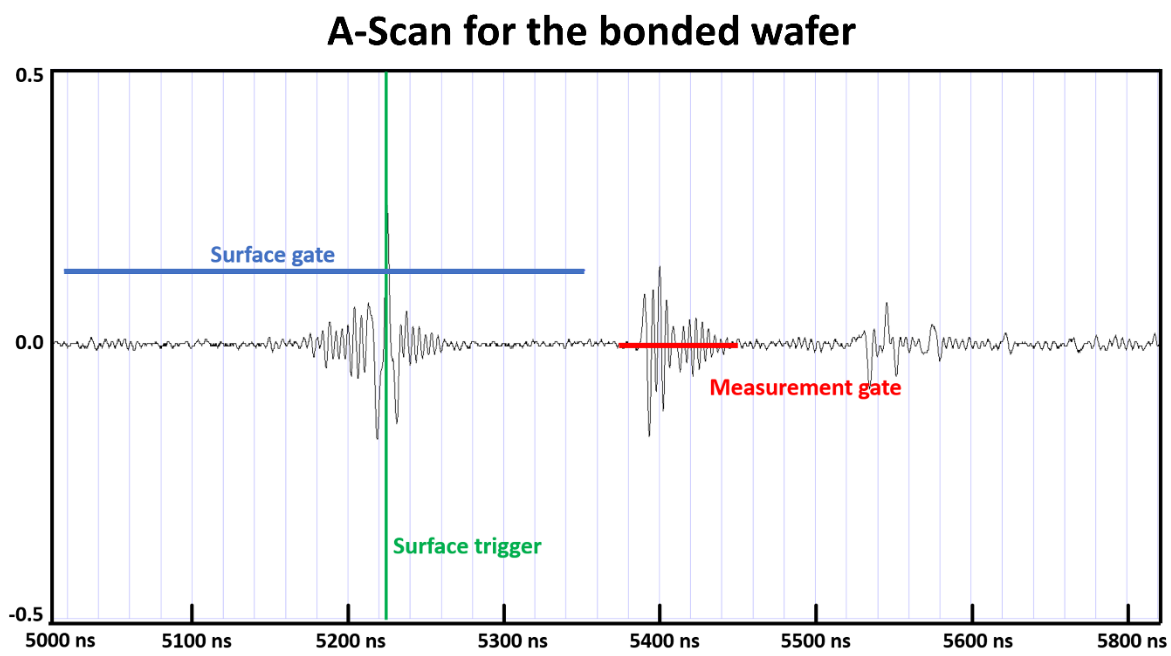

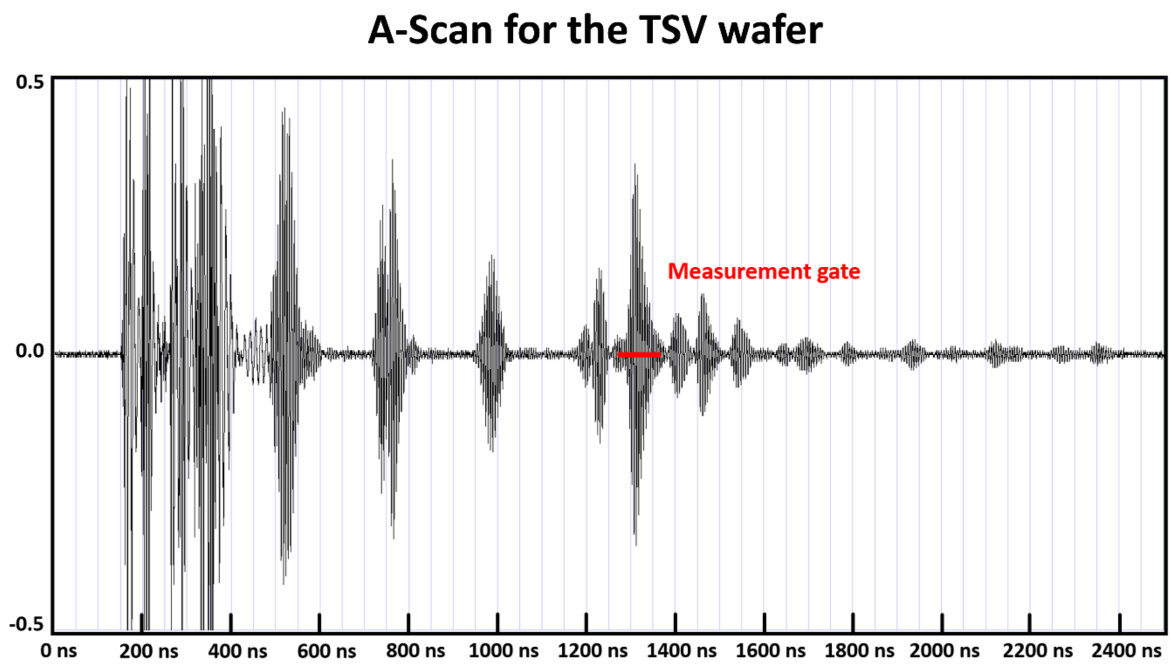

Supplementary Figure 1: A-scans for the bonded and TSV wafer. Gates and surface triggers are indicated in the image. The intensity of the reflected wave ranges from -0.5 to 0.5. Winsam 8.24 software[1] is employed for capturing and preprocessing the A-scans.

SUPPLEMENTARY NOTE 2: Additional metrics for evaluation

In the main text of the paper we defined two additional metrics to complement the PSNR and SSIM values. These two metrics are the edge correlation index (EdgeC) and the count of congruent features found by a SIFT[2] algorithm.

The procedure of calculating EdgeC is presented in Supplementary Figure 2a: First, we use a canny edge detection algorithm (implemented in the scikit-image python package) to find edges in the high-resolution (HR) and super-resolved (SR) image. The detected edges are then put into the normalized correlation function, which outputs a number between -1 and 1. Values close to one indicate that all edges are correlated, i.e. the reconstruction via SR is good. Values close to zero show no correlation at all. Negative outputs define an anti-correlation and may indicate systematic errors in the model.

An example of feature matching with SIFT is shown in Supplementary Figure 2b. Here, the SIFT algorithm implemented in the OpenCV2 python package is used to calculate important features in both the HR and SR image. Then, similar features are matched and the number of matches is counted. The more matches are found, the better the overall reconstruction quality.

Since both metrics employ different hyperparameters which have to be chosen manually, their exact outputs depend on the user. However, the trends in the results (e.g. that machine learning based SR outperforms classical methods) stayed true under all tested hyperparameters. For the final measurements we chose the parameters displayed in Supplementary Table 1. If not mentioned otherwise, the standard parameters of scikit-image and OpenCV2 are kept.

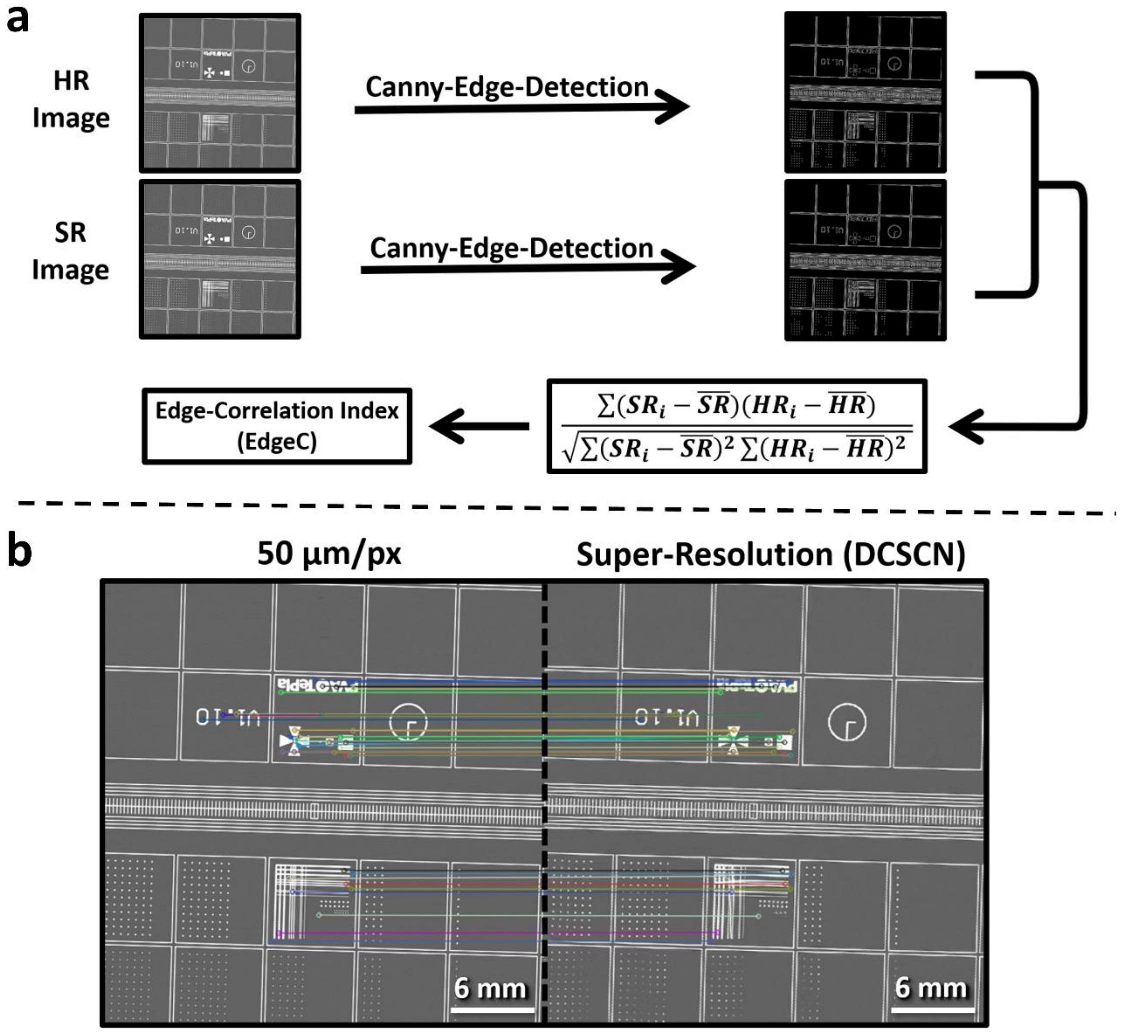

Supplementary Figure 2: Definitions of metrics used in the main text. (a): Definition of the EdgeC (Edge Correlation) metric. First, a canny edge detection algorithm is used to extract the edges from the HR and SR image. Then, the correlation function between those edges is calculated. The sum runs over all pixels. An output value of 1 means a perfect reconstruction of edges whereas a value of 0 indicates that no correlation exists. Negative outputs correspond to anti-correlations. (b): Schematic of our SIFT-based metric. A SIFT algorithm is used to detect congruent features in the high-resolution image and the reconstruction. The more features are found to be similar, the better the reconstruction. Matches are displayed as colored

lines between the 50 µm/px and super-resolution image. Winsam 8.24 software[1] is employed for capturing and preprocessing the C-scan images.

Supplementary Table 1: Hyperparameters used for canny edge detection and SIFT. Parameters which are not mentioned explicitly are kept to the standard values of the employed python packages.

	Low Threshold	*High Threshold*	*nFeatures*	*Contrast Threshold*	*Sigma*
Canny	*0.2*	*0.6*	*-*	*-*	*-*
SIFT	*-*	*-*	*100*	*0.06*	*1*

SUPPLEMENTARY NOTE 3: Evaluation of other SR models on real-world data

In the main text of the paper only the DCSCN and InDI SR models are tested under real-world conditions using a wafer with test structures. In this section, we present a comparison between DCSCN, InDI and the other mentioned SR models under the same conditions. This comparison can be seen in Supplementary Figure 3.

As it is obvious from the images and the metrics, DCSCN performs best under real-world conditions and is closely followed by the SR-GAN model in terms of the metrics. However, when looking at the images, the SR-GAN seems to introduce artefacts including ringing and checkerboard-artefacts. Also, a slight over sharpening is observed. Even though there are no checkerboard artefacts introduced by InDI, the output looks slightly blurrier and is not able to remove jittering (see the blue image). SRResNet, on the other hand, performs poor in general, not even being able to reduce the pixelated nature of the low-resolution input image.

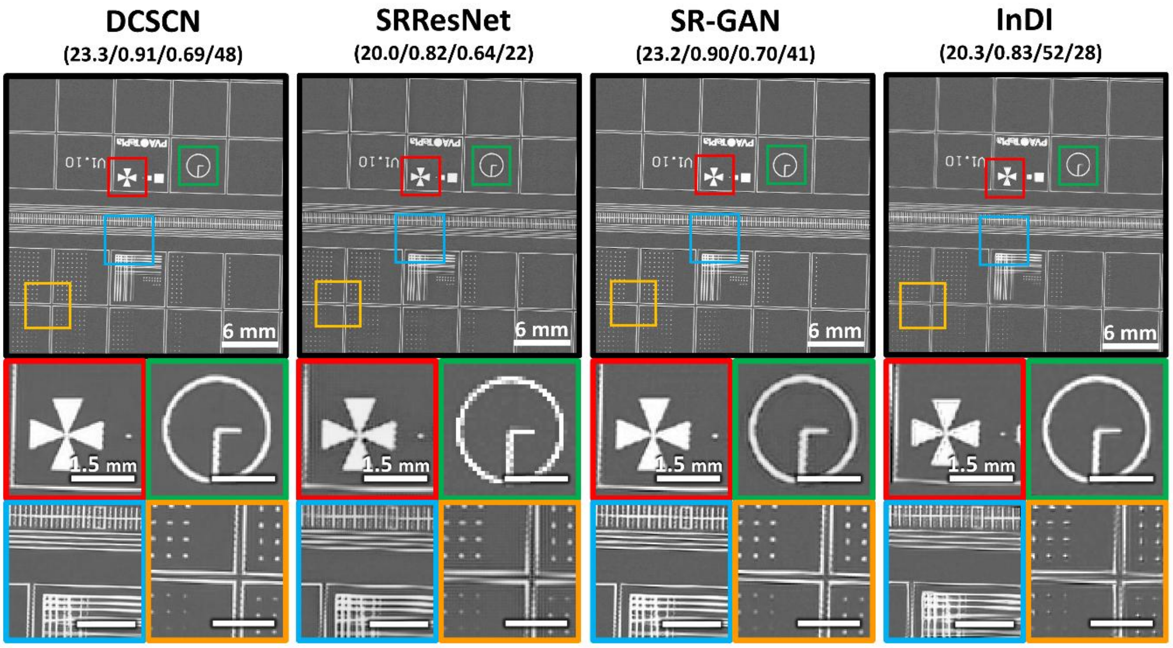

Supplementary Figure 3: Comparison between the different SR models mentioned in the main text. The round brackets contain the metric introduced in the main text: (PSNR, SSIM, EdgeC, SIFT). All metrics show a clear advantage of the DCSCN approach compared to other SR based methods. The images are obtained by upscaling a 100 µm/px C-scan image of the test wafer discussed in the main text. Winsam 8.24 software[1] is employed for capturing and preprocessing the C-scan images.

SUPPLEMENTARY NOTE 4: GAN model architecture

The architecture and training procedure of our SR-GAN implementation is illustrated in Supplementary Figure 4a-c. Details on the architecture and training are given in the main text in the Methods section.

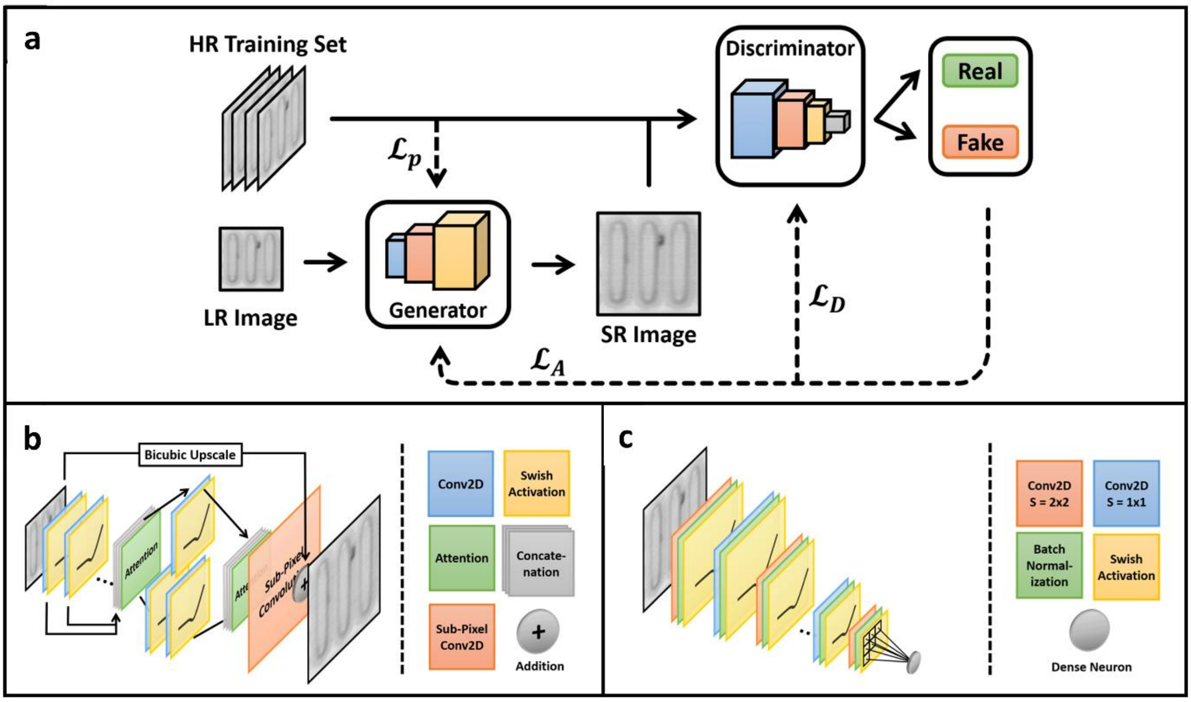

Supplementary Figure 4: Training and model architectures for the GAN generator (DCSCN) and discriminator. (a): Training procedure of SR-GAN. The low-resolution image is passed through the generator. The generated image is then judged by the discriminator to be real or fake and the feedback-loss $\mathcal{L}_A$ is given to the generator. Simultaneously, the generator is constrained by $\mathcal{L}_p$ to be as close to the HR image as possible. $\mathcal{L}_D$ provides information for the discriminator to get better at catching unrealistic images. (b): Model architecture for the generator. The first block consists out of convolutional layers with 176, 160, 144, 128, 112, 96, 80, 64, 48 and 32 filters. The second block (reconstruction block) is split in two. It has convolutional layers with 32 and 32, 64 filters. The kernel size is 3 except for the first layers in the reconstruction block, where we use a kernel size of 1 for feature extraction. (c): Model architecture of the discriminator. We use convolutional layers with strides S = 2, 1, 2, 1, 2, 1, 2. After every strided convolution, the number of filters is doubled, starting with 64 filters in the first convolution. A kernel size of 3 is used for all layers.

SUPPLEMENTARY NOTE 5: Segmentation performance of different model architectures

In the main text it is stated and reasoned that semantic segmentation performance improved when applying super-resolution before labeling and model training. Supplementary Table 2 elaborates on this by showing the segmentation performance of different model architectures. For representativeness, we chose three model architectures often used in scientific applications like material science or medical segmentation. Details on the residual attention u-net can be found in the main text.

The residual u-net presented in the supplementary table 2 has the same architecture as the attention residual u-net presented in the main text, but without attention blocks. The swin u-net was taken from the 'keras-unet-collection' package[3] using a depth of four, num_heads = [4,8,16,32], window_size = 8, patch_size = (2,2) and 8 filters. Note that, since swin is a transformer-based

architecture, training and testing image sizes have to be equal. We therefore split the test image into patches and evaluated each patch.

Supplementary Table 2: Evaluation of segmentation performance for different models. A SAM C-scan image of a wafer is split into two halves for training and testing, respectively. The models were trained on the training data as described in the main text and the area of different structures are evaluated over the whole wafer. All values are given as relative error in percent relative to a manually labeled ground truth of the 50 µm/px C-scan image.

	Residual Att. U-Net			***Residual U-Net***			***SWIN U-Net***		
	50 µm/px	*300 µm/px*	*SR*	*50 µm/px*	*300 µm/px*	*SR*	*50 µm/px*	*300 µm/px*	*SR*
Ion trap recesses	*0.02*	*0.87*	*0.74*	*0.25*	*0.39*	*0.10*	*0.16*	*0.57*	*0.2*
Intact bond	*0.45*	*0.60*	*0.50*	*0.52*	*1.64*	*0.87*	*0.35*	*0.27*	*0.12*
Delaminated bond	*0.23*	*2.32*	*0.21*	*1.59*	*3.51*	*2.09*	*2.2*	*5.36*	*2.05*

SUPPLEMENTARY NOTE 6: Comparison of YOLO and E2E-CNN

As mentioned in the main text, we claim a speed improvement of a factor of 60x for our YOLOv2 model in comparison to the state of the art E2E-CNN approach.[4] In this section, we present the details on how this comparison was done.

The main purpose of E2E-CNN is to localize TSVs and classify them into different defect classes, as can be seen in Supplementary Figure 6 and the original paper. To make a comparison between E2E-CNN and YOLO, we use the original data provided in the E2E-CNN publication. This data is provided by the authors of that paper. We labeled around 4000 images in the proprietary YOLO format. This is less than the 7000 to 20.000 images used for training both CNNs in the original paper. However, every one of our images contains on average 2-3 TSVs, since image tiles of 640x640 are used for training YOLO.

The original E2E-CNN paper states a value of 52s for classifying 96 TSVs, not including localization. To test the speed of YOLO, we utilize the same hardware (NVIDIA Quadro P4000 GPU) and obtain an evaluation time of roughly 1s for localization AND classification of 96 TSVs. This is equivalent to an increase in speed of around 60x when assuming that the localization in the E2E-CNN model takes around 6s. Our model has an accuracy of 96% for classifying TSVs in the right class, which is similar to what the E2E-CNN is capable of.

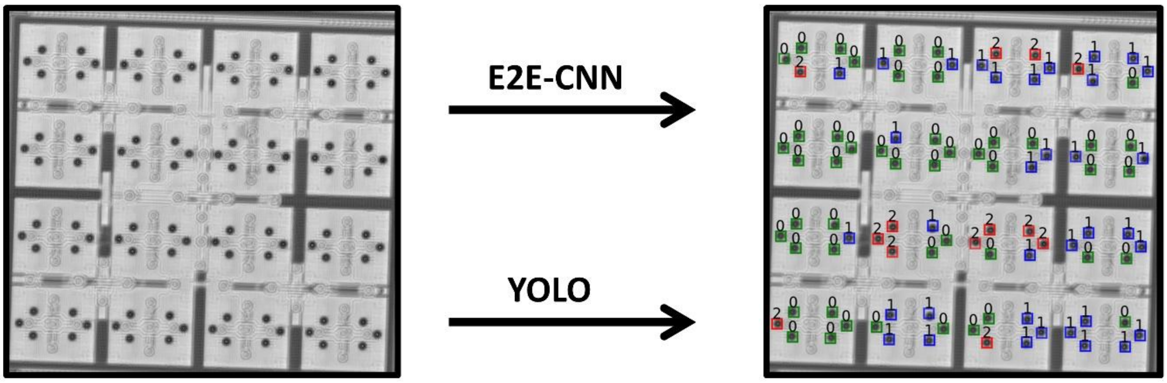

Supplementary Figure 5: TSV samples for our comparison. The E2E-CNN and YOLO object detection algorithms are applied to the same dataset to measure the performance of the models under equivalent conditions. Different colors in the right image correspond to different TSV classes as defined in the original E2E-CNN paper. Winsam 8.24 software[1] *is employed for capturing and preprocessing the C-scan images.*

Referencess

1. PVA TePla. WINSAM 8.24. at https://www.pvatepla-sam.com/en/products/sam-software/.

2. Lowe, D. G. Distinctive Image Features from Scale-Invariant Keypoints. *Int. J. Comput. Vis.* **60**, 91–110 (2004).

3. Sha, Y. Keras-unet-collection. *GitHub repository* at https://doi.org/10.5281/zenodo.5449801 (2021).

4. Paulachan, P., Siegert, J., Wiesler, I. & Brunner, R. An end-to-end convolutional neural network for automated failure localisation and characterisation of 3D interconnects. *Sci. Rep.* **13**, 9376 (2023).
